# Baseline total lesion glycolysis identifies high-risk patients with immunosuppressive signatures in early-stage natural killer/T-cell lymphoma

**DOI:** 10.1093/oncolo/oyaf164

**Published:** 2025-06-24

**Authors:** Xiao Gao, Jie Xiong, Xin-Yun Huang, Hao-Xu Yang, Hui-Juan Zhong, Shu Cheng, Xu-Feng Jiang, Wei-Li Zhao

**Affiliations:** Shanghai Institute of Hematology, State Key Laboratory of Medical Genomics, National Research Center for Translational Medicine at Shanghai, Ruijin Hospital Affiliated to Shanghai Jiao Tong University School of Medicine, Shanghai 200025, China; Shanghai Institute of Hematology, State Key Laboratory of Medical Genomics, National Research Center for Translational Medicine at Shanghai, Ruijin Hospital Affiliated to Shanghai Jiao Tong University School of Medicine, Shanghai 200025, China; Department of Nuclear Medicine, Ruijin Hospital Affiliated to Shanghai Jiao Tong University School of Medicine, Shanghai 200025, China; Shanghai Institute of Hematology, State Key Laboratory of Medical Genomics, National Research Center for Translational Medicine at Shanghai, Ruijin Hospital Affiliated to Shanghai Jiao Tong University School of Medicine, Shanghai 200025, China; Shanghai Institute of Hematology, State Key Laboratory of Medical Genomics, National Research Center for Translational Medicine at Shanghai, Ruijin Hospital Affiliated to Shanghai Jiao Tong University School of Medicine, Shanghai 200025, China; Shanghai Institute of Hematology, State Key Laboratory of Medical Genomics, National Research Center for Translational Medicine at Shanghai, Ruijin Hospital Affiliated to Shanghai Jiao Tong University School of Medicine, Shanghai 200025, China; Department of Nuclear Medicine, Ruijin Hospital Affiliated to Shanghai Jiao Tong University School of Medicine, Shanghai 200025, China; Shanghai Institute of Hematology, State Key Laboratory of Medical Genomics, National Research Center for Translational Medicine at Shanghai, Ruijin Hospital Affiliated to Shanghai Jiao Tong University School of Medicine, Shanghai 200025, China; Pôle de Recherches Sino-Français en Science du Vivant et Génomique, Laboratory of Molecular Pathology, Shanghai 200025, China

**Keywords:** baseline total lesion glycolysis, prognostic marker, early-stage NKTCL, immunosuppressive tumor microenvironment, inflammatory dendritic cells

## Abstract

**Background:**

The post-treatment Deauville scale (DS) and circulating Epstein-Barr virus (EBV)-DNA were used for prediction of long-term remission in natural killer/T-cell lymphoma (NKTCL). However, the baseline biomarkers still remain lacking for clinical application. Here, we hypothesized that ^18^F-FDG PET/CT, as a measure of total tumor burden, would be a baseline biomarker to identify high-risk NKTCL patients.

**Methods:**

We analyzed PET/CT data in early-stage NKTCL patients (*n* = 192) receiving pegaspargase-based regimens from 2 independent clinical trials. The prognostic values of radiomic markers including total lesion glycolysis (TLG), standardized uptake value, and metabolic tumor volume were evaluated in the training (*n* = 127) and validation cohorts (*n* = 65) with a median follow-up of 37 months.

**Results:**

Total lesion glycolysis was a prognosticator of progression-free survival (PFS) and overall survival (OS), with 86.11% and 91.30% sensitivity and 55.77% and 53.25% specificity, respectively, which outperformed the risk model based on posttreatment DS and circulating EBV-DNA (sensitivity 53.85% and specificity 54.24% for PFS, sensitivity 43.75% and specificity 52.34% for OS). Five-year PFS and OS were 92.19% and 96.82% in the low TLG group (<75 g), versus 69.46% and 77.24% in the high TLG group (≥75 g). ScRNA-seq (*n* = 10) and bulk RNA-seq (*n* = 65) data from patients in the trials both revealed that inflammatory dendritic cells, as immunosuppressive signature, were significantly infiltrated in patients with high TLG compared with patients with low TLG.

**Conclusions:**

Baseline TLG reflected an immunosuppressive microenvironment and was an effective radiomic marker for long-term remission in patients with early-stage NKTCL.

**Implications for practice**

Although the clinical outcomes of early-stage Natural killer/T-cell lymphoma (NKTCL) have been remarkably improved with pegaspargase-based regimens, patients refractory to or relapsed from these regimens experienced extremely short survival time, even less than 6 months. Here, we analyzed PET/CT data in 192 early-stage NKTCL patients and identified baseline total lesion glycolysis (TLG) as a prognostic factor for treatment response and long-term survival. Moreover, inflammatory dendritic cells, as immunosuppressive signature, was significantly infiltrated in patients with high TLG. These findings advanced our knowledge on the biological significance of radiomic markers for guiding the precise medicine approaches of NKTCL.

## Introduction

Natural killer/T-cell lymphoma (NKTCL) represents a distinct clinicopathologic entity of aggressive lymphoma and is characterized by persistent infection of Epstein-Barr virus (EBV).^1^ In the past decade, therapeutically targeting aberrant glutamine metabolism with anti-metabolic agent pegaspargase-based regimens, such as MESA (methotrexate, etoposide, dexamethasone, and pegaspargase), P-GemOx (pegaspargase, gemcitabine, and oxaliplatin), GELAD (gemcitabine, etoposide, pegaspargase, and dexamethasone), and ESA (etoposide, dexamethasone, and pegaspargase) in combination with radiotherapy, has significantly improved the clinical outcomes of early-stage (Ann Arbor stage I/II) NKTCL.^2-5^ Risk scoring systems for prognostic evaluation on patients with newly diagnosed NKTCL include the international prognostic index (IPI), prognostic index for natural killer cell lymphoma (PINK), and PINK combined with EBV-DNA (PINK-E). However, effective markers indicating long-term remission in early-stage NKTCL remain to be further investigated.^2,6^

^18^F-fluorodeoxyglucose positron emission tomography/computed tomography (^18^F-FDG PET/CT) scans are routinely used for the initial assessment of total tumor burden in most lymphoma subtypes.^7^ Recently, novel PET-based radiomic markers, including maximum standardized uptake value (SUVmax, the intensity of FDG uptake), metabolic tumor volume (MTV, the total metabolic volumes of all local nodal and extranodal lesions with high metabolic activity of glucose), and total lesion glycolysis (TLG, the volumetric sum adjusted for SUV integrating tumor volume and metabolic activity, which is calculated as the product of MTV and SUVmean),^8^ enable independent predictions on the clinical outcomes of lymphoma patients. The prognostic significance of these parameters varies from lymphoma subtypes. Both MTV and TLG indicate prognosis in Hodgkin lymphoma,^9^ while they are predictive markers for survival in follicular lymphoma^10^ and primary mediastinal (thymic) large B-cell lymphoma,^11^ respectively. Besides, the Deauville scale (DS) based on the SUV is widely applied to evaluate therapeutic response at interim or endpoint posttreatment.^12^ NKTCL patients with complete metabolic response (defined as DS of 1-3) present prolonged survival time compared with those with partial metabolic response (defined as DS of 4-5).^13^ Posttreatment DS in combination with circulating EBV-DNA can predict the risk of treatment failure in NKTCL.^14^ However, baseline radiomic markers with prognostic significance are more helpful for guiding the selection of treatment approaches.^15^

In the biological setting, ^18^F-FDG uptake is correlated with glucose metabolism and the expression of glucose regulatory genes *GLUT1*, *ALDOA*, *FBP1*, *HIF1A*, *VEGF*, *MVD*, and *TP53* by tumor cells.^16-18^ More recently, with increasing knowledge of tumor microenvironment, metabolic alterations have been recognized not only as the characteristics of the tumor compartment but also the tumor-infiltrating immune cells.^19^ For example, SUVmax links to CD8+ tumor-infiltrating lymphocytes, CD163+ tumor-associated macrophages (TAMs), and Foxp3+ regulatory T cells.^20^ This encouraged us to investigate the biological significance of radiomic markers, and the associated molecular signatures underlying disease progression in early-stage NKTCL.

In the present study, we identified the value of baseline metabolic parameters for therapeutic response and disease prognosis using 2 cohorts from clinical trials (NCT02825147^2^ and NCT02631239^5^) of early-stage NKTCL coordinated by the Multicenter Hematology-Oncology Programs Evaluation System (M-HOPES) network from China. The M-HOPES is a collaborative research network for multicenter clinical trials, which includes major clinical centers and hospitals in China. Meanwhile, the relationship between metabolic markers and biological signatures was analyzed in NKTCL at single-cell resolution.

## Methods

### Study population

Newly diagnosed early-stage NKTCL patients (*n* = 249) receiving pegaspargase-based regimens plus sandwiched radiotherapy with qualified ^18^F-FDG PET/CT scan data were enrolled. Patients were originally included in 2 individual clinical trials NCT02825147 (also known as NHL-004, *n* = 153)^2^ and NCT02631239 (*n* = 38),^5^ as well as a real-world cohort (*n* = 58). The trial NCT02825147 (March 2016 to July 2020) is a prospective, multicenter, randomized study to compare the efficacy and safety of ESA and MESA in combination with sandwiched radiotherapy as a first-line treatment for newly diagnosed early-stage NKTCL. The trial NCT02631239 (September 2013 to October 2015) is a phase II trial of MESA sandwiched with radiotherapy to evaluate its therapeutic efficacy and safety in newly diagnosed, early-stage NKTCL. The real-world cohort (July 2020 to November 2022) included 50 patients treated with ESA sandwiched with radiotherapy according to the protocol of NHL-004 and 8 patients treated with regimen containing PD-1 antibody. Sandwiched radiotherapy was performed on 21-35 days after 2 cycles for the involved local focus at a dose of 50 Gy in all patients.^2,5^

The inclusion criteria were as follows: (1) all patients were pathologically confirmed as NKTCL according to WHO classification criteria and categorized as stage I or II according to the Ann Arbor staging system; (2) the induction treatment was pegaspargase-based regimens; and (3) Digital Imaging and Communications in Medicine (DICOM) files of PET/CT scan pre- or posttreatment were available. Exclusion criteria were as follows: (1) regimens containing programmed cell death protein 1 (PD1) antibody (*n* = 8); (2) DICOM files of baseline PET/CT not available (*n* = 27); (3) no qualified PET/CT imaging (*n* = 22), such as incomplete whole body ^18^F-FDG PET/CT scan data or missed essential DICOM information.

Single-cell RNA-seq (scRNA-seq) analyses were performed in 10 NKTCL patients with qualified fresh tumor biopsy samples. Bulk RNA-seq analyses were performed in 65 NKTCL patients. Whole genome sequencing (WGS), whole exome sequencing (WES), and targeted sequencing were performed in 85, 27, and 107 NKTCL patients, respectively. Individual trials, the use of imaging data, and transcriptomic data were approved by the institutional review board and all patients provided written informed consent.

### Transcriptomic and genomic sequencing

The preparation of the single-cell suspensions, synthesis of complementary DNA, and construction of single-cell libraries were performed using a Chromium single cell 5′ kit v2 (10× Genomics), which were sequenced on MGI-2000 sequencer with 150bp pair-end reads.^21^ mRNA/cDNA Library construction was performed with TruSeq RNA Sample Prep Kit and sequenced on Illumina Hiseq X10.^22^ DNA libraries of WGS and WES were prepared with TruSeq Nano DNA Sample Prep Kit (Illumina) and SureSelect capture library Kit (Agilent), respectively. Paired-end sequencing was performed using Illumina NovaSeq 6000 System.^22^

### ^18^F-FDG PET/CT

^18^F-FDG PET/CT scans were performed using a Discovery VCT system (GE Healthcare, USA). Patients were required to fast for at least 6 hours before examination, and blood glucose was kept under 7.0 mmol/L. PET/CT scans were taken from the head to thigh after intravenous administration of 5-6 MBq of ^18^F-FDG per kilogram of body weight about 1 hours. The CT scan data were collected with 120-180 mAs, 140 kV, and a gantry rotation speed of 0.8 s. Integrated PET and CT images were obtained on Xeleris (GE Healthcare, Chicago, IL) and Advantage workstation (GE Healthcare, Chicago, IL).

Raw data of ^18^F-FDG PET/CT images were collected in DICOM format and reviewed by 2 independent senior nuclear medicine physicians, who were blinded to patient outcomes. Delineations were performed by trained researchers. Volumes of interest (VOIs) of PET/CT scans were segmented with a semi-automated workflow (SyngoVia software; multifoci segmentation tool; Siemens). The SUV of VOIs was obtained automatically after manually excluding physiological uptake. SUVmax was defined as the uptake in the brightest pixel in the VOI. MTV was defined as the total volume of the metabolically active tumor in the VOI. According to previous studies,^23,24^ the 41% SUVmax threshold method was used for MTV and TLG computation. TLG was calculated as the product of MTV and SUVmean.

### Statistical analysis

The chi-square test or Fisher’s exact test was applied to identify associations between categorical variables. The nonparametric 2-sided Wilcoxon or Mann–Whitney test was used for the comparison of the two groups. One-way ANOVA or Brown–Forsythe test was used for comparisons among multiple groups. Progression-free survival (PFS) was calculated from the date of enrollment to the date of disease progression or death from any cause, and overall survival (OS) was calculated from the date of enrollment to the final follow-up or death from any cause. Survival analysis was calculated by using Kaplan–Meier estimates, and a comparison between categories was made by the log-rank test. Cox regression analysis was used for multivariable analysis of survival outcomes and classified a two-sided *P* value less than .05 as significant. Spearman correlation was used for analyzing the association of 2 continuous variables. Statistical analysis was performed with the SPSS software (version 29). Negative predictive values (NPVs) and positive predictive values (PPVs) were calculated using the online MedCalc program (MedCalc Software Ltd. https://www.medcalc.org/calc/diagnostic_test.php, Version 22.023).

## Results

### Patient characteristics

**Figure 1.**
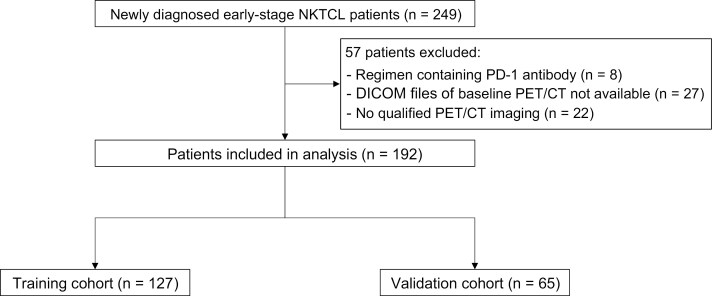
Study flow diagram. A total of 249 patients with newly diagnosed early-stage NKTCL were available in the M-HOPES database, of whom 192 were included in this study and designated as training cohort (*n* = 127) and validation cohort (*n* = 65).

As shown in Figure 1, 249 newly diagnosed patients with early-stage NKTCL were screened, with 192 patients from 11 clinical centers included for further analysis. Considering that large multicenter randomized prospective phase III clinical trials provide high-quality standardized data and are commonly used for biomarker model development,^25,26^ we therefore referred patients from the trial NHL-004 (NCT02631239) as the training cohort (*n* = 127). Patients from the trial NCT02825147 and the real-world cohort were referred to as the validation cohort (*n* = 65). All patients include in this study were nasal type disease. Baseline characteristics of all patients are listed in Table 1, revealing no significant difference between the training and validation cohorts. Besides, there was no significant difference in the PFS and OS between the training and validation cohorts (Supplementary Figure 1).

**Table 1.** Patient characteristics.

		Total (*n* = 192)	Training cohort (*n* = 127)	Validation cohort (*n* = 65)	*P*
Sex					.052
	Male	138 (72)	97 (76)	41 (65)	
	Female	54 (28)	30 (24)	24 (35)	
Age					.741
	≤60	154 (80)	101 (80)	53 (82)	
	>60	38 (20)	26 (20)	12 (18)	
Performance status					.719
	ECOG 0 or 1	184 (96)	121 (95)	63 (97)	
	ECOG ≥ 2	8 (4)	6 (5)	2 (3)	
Ann Arbor stage					.349
	I	121 (63)	83 (65)	38 (58)	
	II	71 (37)	44 (35)	27 (42)	
Serum lactate dehydrogenase					.659
	Normal	108 (56)	70 (55)	38 (58)	
	Increased	84 (44)	57 (45)	27 (42)	
B symptoms					.641
	Absent	115 (60)	78 (61)	37 (57)	
	Present	77 (40)	49 (39)	28 (43)	
Lymph node involvement					.912
	Absent	126 (66)	83 (65)	43 (66)	
	Present	66 (34)	44 (35)	22 (34)	
Baseline Epstein-Barr virus DNA					.708
	Lower than detection limit	114 (60)	77 (61)	37 (58)	
	Detected	77 (40)	50 (39)	27 (42)	
IPI risk					.793
	Low (0-1)	175 (91)	115 (91)	60 (92)	
	Low-intermediate (2)	17 (9)	12 (9)	5 (8)	
	High-intermediate (3)	0 (0)	0 (0)	0 (0)	
	High (4-5)	0 (0)	0 (0)	0 (0)	
PINK risk					.511
	Low (0)	150 (78)	101 (80)	49 (75)	
	Intermediate (1)	42 (22)	26 (20)	16 (25)	
	High (≥2)	0 (0)	0 (0)	0 (0)	
PINK-E risk					.516
	Low (0-1)	171 (90)	115 (91)	56 (88)	
	Intermediate (2)	20 (10)	12 (9)	8 (12)	
	High (≥3)	0 (0)	0 (0)	0 (0)	

*P* were compared between training cohort and validation cohort by χ2 test or Fisher’s exact test.

Abbreviations: ECOG, eastern cooperative oncology group; IPI, international prognostic index; PINK, prognostic index of natural killer lymphoma; PINK-E, PINK combined with Epstein-Barr virus DNA.

With a median follow-up of 37.25 (range 0.70 to 94.57) months, the 5-year PFS and OS were 79.58% and 86.74%, respectively. Univariate Cox analysis revealed that none of the clinical parameters was associated with PFS (Supplementary Figure 2A) or OS (Supplementary Figure 2B). For IPI (Supplementary Figure 2C) or PINK (Supplementary Figure 2D), no significant differences in PFS and OS were detected among the risk groups. For PINK-E (Supplementary Figure 2E), the intermediate-risk group was associated with inferior PFS (*P* = .021), but not OS (*P* = .111), as compared with the low-risk group.

### Baseline PET metabolic parameters

**Figure 2.**
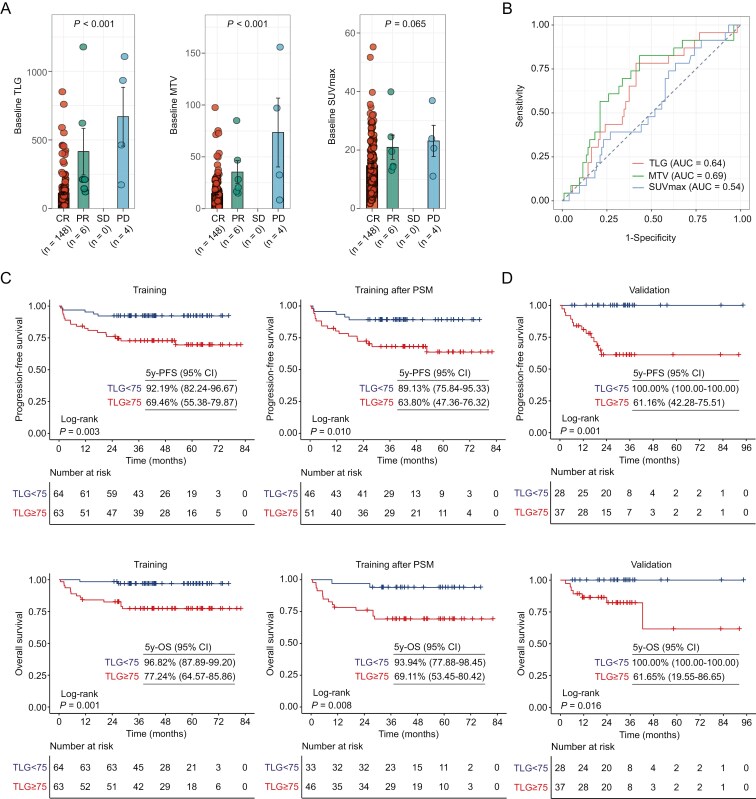
Baseline PET metabolic parameters. (A) Baseline PET metabolic parameters TLG, MTV, and SUVmax in the patients with complete remission (CR, *n* = 148), partial remission (PR, *n* = 6), stable disease (SD, *n* = 0), and progressive disease (PD, *n* = 4) at endpoint post-treatment. *P* values were compared using ANOVA. Data were represented as mean ± SEM. (B) Receiver operating characteristic curves showed the area under the curve of baseline TLG, MTV, and SUVmax according to PFS. (C and D) PFS and OS according to baseline TLG with a cutoff of 75 g in the training cohort, training cohort after propensity-score matching (C), and validation cohort (D). *P* values were calculated by log-rank test.

Baseline PET metabolic parameters TLG, MTV, and SUVmax were first compared according to therapeutic response (Figure 2A). The representative images with low or high tumor burden were presented in Supplementary Figure 3. TLG and MTV were significantly higher in patients with progressive disease than those with complete or partial remission, which were subjected for further analysis. SUVmax showed no difference according to treatment response. The median baseline TLG and MTV were 77.10 g (range 4.47-1177.86 g) and 11.15 cm^3^ (range 0.98-155.79 cm^3^), respectively. To determine the best cutoff value, we choose PFS as the endpoint to analyze the receiver operating characteristic and identified that the area under the curve was 0.64 for TLG (*P* = .019) and 0.69 for MTV (*P* = .003, Figure 2B), respectively. The thresholds for TLG (75 g) and MTV (10 cm^3^) were selected as the optimal cutoff value for PFS and OS in the training and training after propensity-score matching cohort (Figure 2C and Supplementary Figure 4A). The negative prognostic impact of high TLG was further revealed in the validation cohort (Figure 2D). The 5-year PFS for patients with low TLG (*n* = 28) and high TLG (*n* = 37) was 100.00% and 61.16%, respectively (*P* = .001). The 5-year OS for patients with low and high TLG were 100.00% and 61.65%, respectively (*P* = .016). However, no significant differences in PFS (*P* = .225) and OS (*P* = .731) were observed in the validation cohort between the low MTV (*n* = 25) and high MTV (*n* = 40) group (Supplementary Figure 4B). Besides, high baseline TLG was associated with Ann Arbor stage II (*P* = .025), B symptoms (*P* = .012), increased serum lactic dehydrogenase (*P* = .001), and baseline EBV-DNA copies number (*P* = .010), as compared with low baseline TLG (Supplementary Table 1).

**Figure 3.**
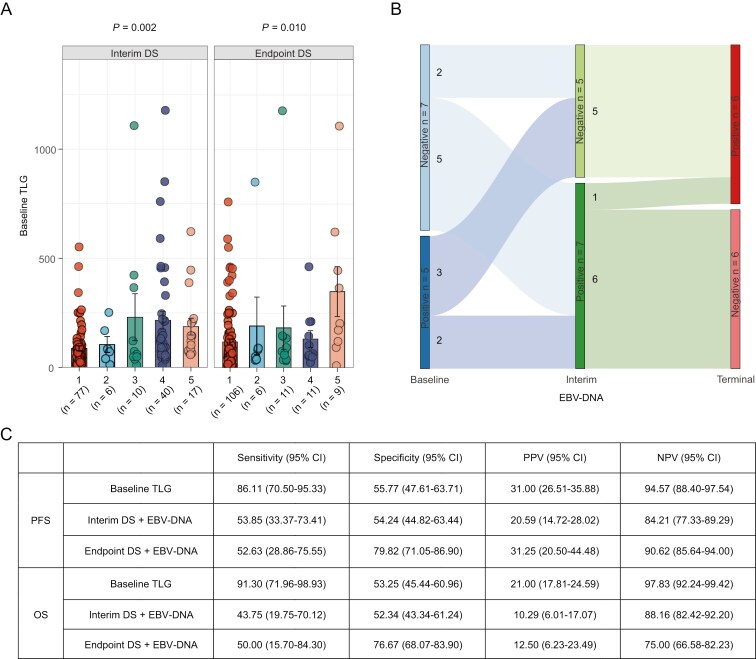
Predictive value of baseline TLG. (A) Comparison of baseline TLG according to DS 1-5 at interim (*n* = 150) or endpoint (*n* = 143) posttreatment. *P* values were compared using ANOVA. Data were represented as mean ± SEM. (B) Comparison of dynamic circulating EBV-DNA copies number at baseline, interim, and endpoint in TLG low (<75 g, *n* = 65) and high (≥75 g, *n* = 61) group. *P* values between-group comparisons were compared using the Mann–Whitney test; *P* values in group comparisons were compared using the Wilcoxon test. (C) Prognostic power of baseline TLG, interim DS + EBV-DNA, and endpoint DS + EBV-DNA according to PFS and OS, respectively.

Based on the previous report that posttreatment DS on PET/CT and circulating EBV-DNA were important indicators for therapeutic response in NKTCL,^14^ we compared TLG according to interim or endpoint DS, revealing significantly higher levels of baseline TLG in DS 3-4 at interim evaluation and DS 5 at endpoint evaluation than those of DS 1-2 at interim evaluation and DS 1-4 at endpoint evaluation, respectively (Figure 3A). Although circulating EBV-DNA remarkably declined at interim and endpoint evaluation upon treatment, we found that patients positive for circulating EBV-DNA at interim or final evaluation were mainly from high TLG group (*n* = 8, 67%, Figure 3B).

To clarify the predictive performance of TLG, we further compared the predictive value of TLG with a previously reported risk model based on posttreatment DS and circulating EBV-DNA at diagnosis.^14^ Baseline TLG showed superior sensitivity (86.11% for PFS and 91.30% for OS) and NPV (94.57% for PFS and 97.83% for OS), and comparable specificity (55.77% for PFS and 53.25% for OS) and PPV (31.00% for PFS and 21.00% for OS), as compared with the risk model at interim or endpoint evaluation, as well as MTV (Supplementary Figure 4C). Taken together, baseline TLG was an effective prognostic indicator in early-stage NKTCL.

### Biological signatures of TLG

**Figure 4.**
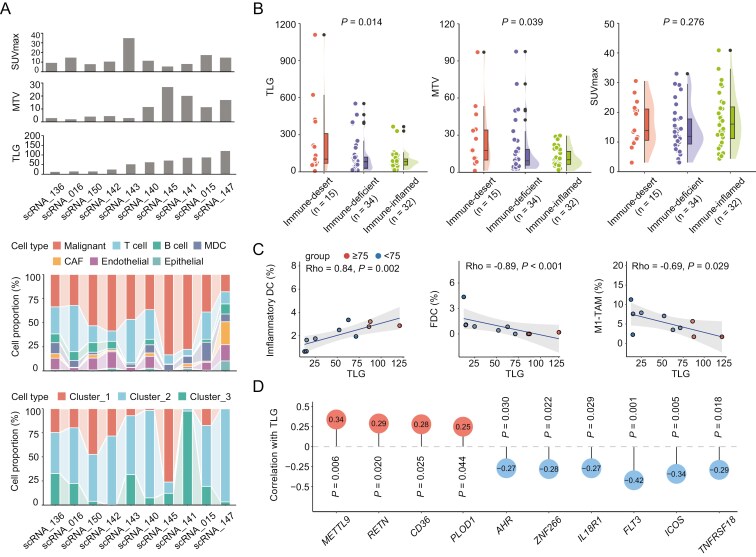
Baseline TLG and tumor microenvironment. (A) Correlations of baseline TLG, MTV, and SUVmax and indicated cell types and the malignant cell-of-origins based on scRNA-seq data of NKTCL patients (*n* = 10). (B) Comparison of baseline TLG, MTV, and SUVmax according to the immune subtypes based on 81 NKTCL patients. *P* values were calculated using ANOVA. Data were represented as mean ± SEM. (C) Correlation of baseline TLG with inflammatory DC, FDC, and M1-TAM based on scRNA-seq data of NKTCL patients (*n* = 10). (D) Correlation of baseline TLG with indicated genes associated with inflammatory DC and immune checkpoints based on bulk RNA-seq data of NKTCL patients (*n* = 65).

To characterize the biological features of baseline TLG, we retrieved differentially expressed genes using RNA-seq data between high TLG (*n* = 39) and low TLG (*n* = 26) patients and proteins using proteomic data between high TLG (*n* = 44) and low TLG (*n* = 33) patients,^21^ respectively. A total of 239 upregulated genes and 352 downregulated genes, as well as 284 upregulated proteins and 324 downregulated proteins were observed (Supplementary Figure 5A). Enrichment analysis showed that differentiated genes and proteins were mainly enriched with metabolic and immune pathways (Supplementary Figure 5B). Considering the complexity of tumor microenvironment, we performed integrative analysis using tumoral scRNA-seq data of 10 newly diagnosed NKTCL patients^21^ with available PET/CT results (Figure 4A). Radiomic markers showed no correlation with either the proportion of the malignant cells or the cell-of-origins (Supplementary Figure 5C and D). Besides, the mutational pattern was quite similar (Supplementary Figure 5E) according to TLG, MTV, and SUVmax, based on previously defined 5 functional clusters of NKTCL, namely RNA helicase family, tumor suppressors, JAK-STAT pathway, epigenetic modifiers, and RAS-MAPK pathway.^22^ We next compared the baseline TLG, MTV, and SUVmax level according to molecular subtypes^22^ and found no significant difference among TSIM (*n* = 49), HEA (*n* = 15), and MB (*n* = 17) subtypes (Supplementary Figure 5F). EBV predominantly infected tumor cells at single-cell resolution with high expression of viral genes *BALF3*, *BALF4*, *BALF5*, *BILF1*, *BNLF2a*, *BNLF2b*, and *LMP1*.^21^ Neither tumor EBV percentage nor viral gene expression were correlated with radiomic markers (Supplementary Figure 5G and H). These data encouraged further investigation into the tumor-infiltrating immune cells.

According to a previously defined 3 microenvironment phenotypes based on distinct compositions 36 tumor-infiltrating immune cell types in NKTCL,^21^ the immune-desert subtype (*n* = 15) showed significantly higher TLG (median = 103.10, *P* = .014) and MTV (median = 17.70, *P *= .039) levels than that of the immune-deficient subtype (*n* = 34, median TLG = 82.76) and immune-inflamed subtype (*n* = 32, median TLG = 82.35), indicating baseline TLG and MTV were significantly associated with lymphoma microenvironment (Figure 4B). TLG was positively correlated with immune suppressive inflammatory dendritic cell (DC) (Rho = 0.84, *P* = .002), negatively correlated with immune-activated follicular DC (FDC) (Rho = −0.89, *P* < .001) and M1-TAM (Rho = −0.69, *P* = .029) (Figure 4C and Supplementary Figure 6). As confirmed using bulk RNA-seq data (*n* = 65), inflammatory DC marker genes^27^*METTL9* (Rho = 0.34, *P* = .006), *RETN* (Rho = 0.29, *P* = .020), *CD36* (Rho = 0.28, *P* = .025), and *PLOD1* (Rho = 0.25, *P* = .044) were positively correlated with TLG (Figure 4D). Meanwhile, inflammatory DC putative transcriptional suppressor *AHR* (Rho = −0.27, *P* = .030) and *ZNF266* (Rho = −0.28, *P* = .022), as well as DC development and maturation regulator^27,28^*IL18R1* (Rho = −0.27, *P* = .029) and *FLT3* (Rho = −0.42, *P* = .001), were negatively correlated with TLG. Immune stimulating checkpoints *ICOS* (Rho = −0.34, *P* = .005) and *TNFRSF18* (Rho = −0.29, *P* = .018) were also negatively correlated with TLG (Figure 4D). Taken together, high TLG may reflect an immunosuppressive signature with tumor infiltration with inflammatory DCs in NKTCL, rendering them resistant to anti-metabolic treatment. The 13 refractory/relapsed patients with high TLG received second-line regimens, including anti-PD1 antibody and P-GemOx (*n* = 4), anti-PD1 antibody and GemOx (*n* = 3), anti-PD1 antibody and Chidamide (*n* = 1), anti-PD1 antibody and Pegasapargase (*n* = 1), SMILE (*n* = 2), GLIDE (*n* = 1), and P-GemOx (*n* = 1). Of note, anti-PD1 antibody-containing regimens significantly prolonged the survival time, comparing with chemotherapies alone (Supplementary Figure 7). The median survival time since disease progression was not reached and 13.7 months for anti-PD1 antibody-containing regimens and chemotherapies, respectively. Therefore, anti-PD1 antibody could overcome the poor prognostic features associated with high TLG.

## Discussion

Based on the training and validation cohort from prospective clinical trials with long-term follow-up, we demonstrated that high baseline TLG (≥75 g) was significantly associated with poor clinical outcomes of early-stage NKTCL patients in the era of anti-metabolic treatment.

Identifying prognostic markers at diagnosis is important for response prediction and treatment decision. Baseline TLG is easy to use and can distinguish patients from durable remission to early progression. In our study, despite the overall improvement of survival with current pegaspargase-based regimens (5-year PFS 78.56% and 5-year OS 84.70%), the 5-year PFS and OS varied substantially (from 100% to 60%) when stratified by the baseline TLG. More importantly, baseline TLG, reflecting overall tumor burden,^8^ correlated with post-treatment DS, indicating potential resistance to pegaspargase-based regimens. Novel agents, including immune checkpoint inhibitors, signaling pathway inhibitors, epigenetic targeted agents, etc., in combination with chemotherapy are necessary to enhance therapeutic efficacy.^29^ Therefore, baseline TLG was a useful index for risk stratification and better selection of treatment strategies in future design of prospective trials in early stage NKTCL.

Tumor cells increase the utilization of glucose and glutamine, exhibiting aerobic glycolysis and leading to growth advantage,^30^ and EBV infection also enhances aerobic glycolysis and contributes to tumor progression.^31^ Metabolic alterations are the oncogenic hallmark of NKTCL, characterized by enhanced glutamine addiction^32^ and virion production of EBV.^33^ In pancreatic cancer, glutamine and associated glutamate showed inverse correlation with TLG.^34^ In this study of NKTCL, baseline TLG, as calculator of metabolic activity,^8^ correlated with dynamic changes of circulating EBV-DNA, underlying the therapeutic potential of EBV-targeted strategy in patients resistant to pegaspargase.

Immune cell infiltration within the tumor microenvironment determines clinical outcomes in multiple cancers.^35,36^ Of note, high baseline TLG can identify a subset of patients with remarkably poor prognosis, characterized by immunosuppressive status with tumor infiltration of inflammatory DC, instead of FDC and M1-TAM. We previously reported immune alterations during EBV-induced lymphomagenesis in NKTCL and defined 3 major immune subtypes, namely immune-desert, -deficient, and -inflamed.^21^ Patients of immune-desert subtype, characterized by complete suppression of innate and adaptive immunity, present with high baseline TLG. Similarly in another study of NKTCL, LMP1+ malignant NK cells exhibited the most intensive immunosuppressive interaction of CD86-CTLA4 and PDL1-PD1 with T cells, correlating with unfavorable prognosis.^37^ Here, we uncovered that baseline TLG was closely related to the lymphoma microenvironment and immune status in NKTCL. Interestingly, in our recently reported clinical trial of pegaspargase plus anti-PD1 antibody sintilimab in treating NKTCL,^38^ there was no difference in TLG between the responders and nonsresponders. Therefore, the immunosuppressive signatures of high TLG could be overcome by anti-PD1 antibody in NKTCL.

The study had limitations. Despite the prognostic value of TLG validated by systematic and effective methods, it remained to be validated in different populations and treatment approaches. Besides, the cutoff value for TLG could be varied in different studies due to segmentation methods,^39^ it will be of great interest to explore artificial intelligence aided TLG analysis in future studies, which will provide standardized and generalized procedures for different researches.

In conclusion, TLG measured on baseline PET was an effective prognostic marker in early-stage NKTCL treated with pegaspargase-based regimens. These findings also advanced our knowledge on the biological significance of radiomic markers for guiding the precise medicine approaches of NKTCL.

## Supplementary Material

oyaf164_suppl_Supplementary_Figures_S1-S7_Tables_S1

## Data Availability

Previously published WES/WGS/Targeted sequencing data, RNA-seq data, and scRNA-seq data reanalyzed here were included in bibliographic references and available from NODE (http://www.biosino.org/node) by pasting the accession code OEP000498 and OEP003404. All other data supporting the findings of this study are available from the corresponding authors upon reasonable request.
