# Supplementary material for: Baseline total lesion glycolysis identifies high-risk patients with immunosuppressive signatures in early-stage natural killer/T-cell lymphoma
Source: Oncologist. 2025 Jun 24;30(6):oyaf164. doi: 10.1093/oncolo/oyaf164 (PMC12199695; doi:10.1093/oncolo/oyaf164)
Supplement: oyaf164_suppl_Supplementary_Figures_S1-S7_Tables_S1 [file oyaf164_suppl_supplementary_figures_s1-s7_tables_s1.docx]

**Baseline total lesion glycolysis identifies high-risk patients with immunosuppressive signatures in early-stage natural killer/T-cell lymphoma**

**Xiao Gao^1,*^, Jie Xiong^1,*,#^, Xin-Yun Huang^2,*^, Hao-Xu Yang^1^, Hui-Juan Zhong^1^, Shu Cheng^1^, Xu-Feng Jiang^2^, Wei-Li Zhao^1,3,#^**

1 Shanghai Institute of Hematology, State Key Laboratory of Medical Genomics, National Research Center for Translational Medicine at Shanghai, Ruijin Hospital Affiliated to Shanghai Jiao Tong University School of Medicine, Shanghai, China

2 Department of Nuclear Medicine, Ruijin Hospital Affiliated to Shanghai Jiao Tong University School of Medicine, Shanghai, China

3 Pôle de Recherches Sino-Français en Science du Vivant et Génomique, Laboratory of Molecular Pathology, Shanghai, China

* These authors contributed equally.

# Correspondence to: Wei-Li Zhao, Email: zhao.weili@yahoo.com; Jie Xiong, Email: xiongjie_sih@163.com; Shanghai Institute of Hematology, State Key Laboratory of Medical Genomics, National Research Center for Translational Medicine at Shanghai, Ruijin Hospital Affiliated to Shanghai Jiao Tong University School of Medicine, Shanghai, China, Tel: 0086-21-64370045; Fax: 0086-21-64743206.

**Supplementary Materials**

**Supplementary Table S1. Correlations between clinical indices and baseline TLG**

|  |  | Baseline TLG (n=192) | | *P* |
| --- | --- | --- | --- | --- |
|  |  | <75 g | ≥75 g |  |
| Sex |  |  |  | *0.025 |
|  | Male | 62 (65) | 76 (79) |  |
|  | Female | 34 (35) | 20 (21) |  |
| Age |  |  |  | 1.000 |
|  | ≤60 | 77 (80) | 77 (80) |  |
|  | >60 | 19 (20) | 19 (20) |  |
| Performance status |  |  |  | 0.279 |
|  | ECOG 0 or 1 | 94 (98) | 90 (94) |  |
|  | ECOG ≥2 | 2 (2) | 6 (6) |  |
| Ann Arbor stage |  |  |  | *0.025 |
|  | I | 68 (71) | 53 (55) |  |
|  | II | 28 (29) | 43 (45) |  |
| Serum lactate dehydrogenase |  |  |  | *0.001 |
|  | Normal | 65 (68) | 43 (45) |  |
|  | Increased | 31 (32) | 53 (55) |  |
| B symptoms |  |  |  | *0.012 |
|  | Absent | 66 (69) | 49 (51) |  |
|  | Present | 30 (31) | 47 (49) |  |
| Lymph node involvement |  |  |  |  |
|  | Absent | 69 (72) | 57 (59) | 0.068 |
|  | Present | 27 (28) | 39 (41) |  |
| Baseline Epstein-Barr virus DNA |  |  |  |  |
|  | Lower than detection limit | 66 (69) | 48 (50) | *0.010 |
|  | Detected | 30 (31) | 47 (50) |  |
| IPI risk |  |  |  | 0.204 |
|  | Low (0-1) | 90 (94) | 85 (89) |  |
|  | Low-intermediate (2) | 6 (6) | 11 (11) |  |
|  | High-intermediate (3) | 0 (0) | 0 (0) |  |
|  | High (4-5) | 0 (0) | 0 (0) |  |
| PINK risk |  |  |  | 0.727 |
|  | Low (0) | 76 (79) | 74 (77) |  |
|  | Intermediate (1) | 20 (21) | 22 (23) |  |
|  | High (≥2) | 0 (0) | 0 (0) |  |
| PINK-E risk |  |  |  | 0.149 |
|  | Low (0-1) | 89 (93) | 82 (86) |  |
|  | Intermediate (2) | 7 (7) | 13 (24) |  |
|  | High (≥3) | 0 (0) | 0 (0) |  |

**Supplementary Figures**

**
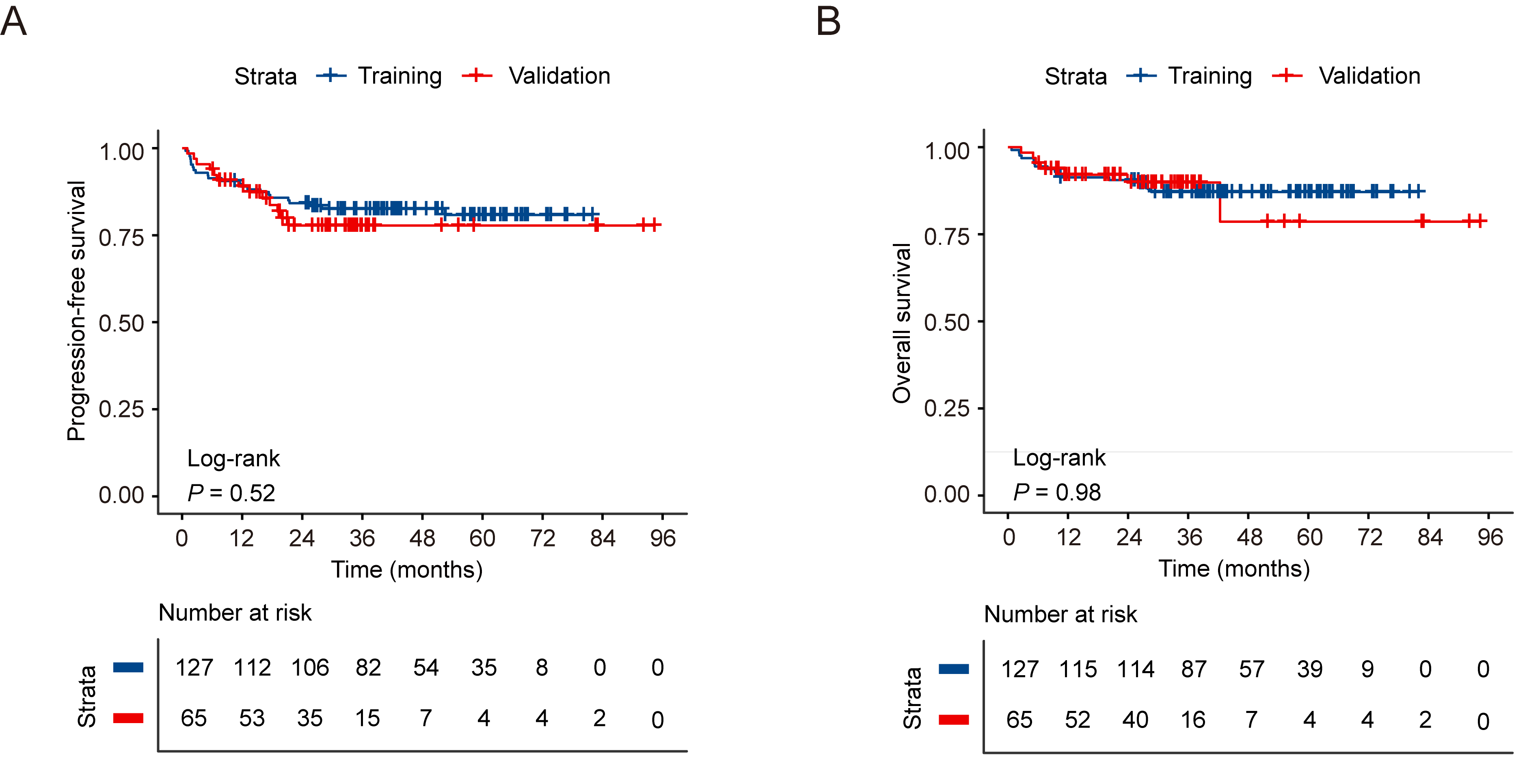
**

**Supplementary Figure S1. Survival analysis in the training and validation cohorts.**

PFS (A) and OS (B) in the training and validation cohorts. *P* values were calculated by log-rank test.

**
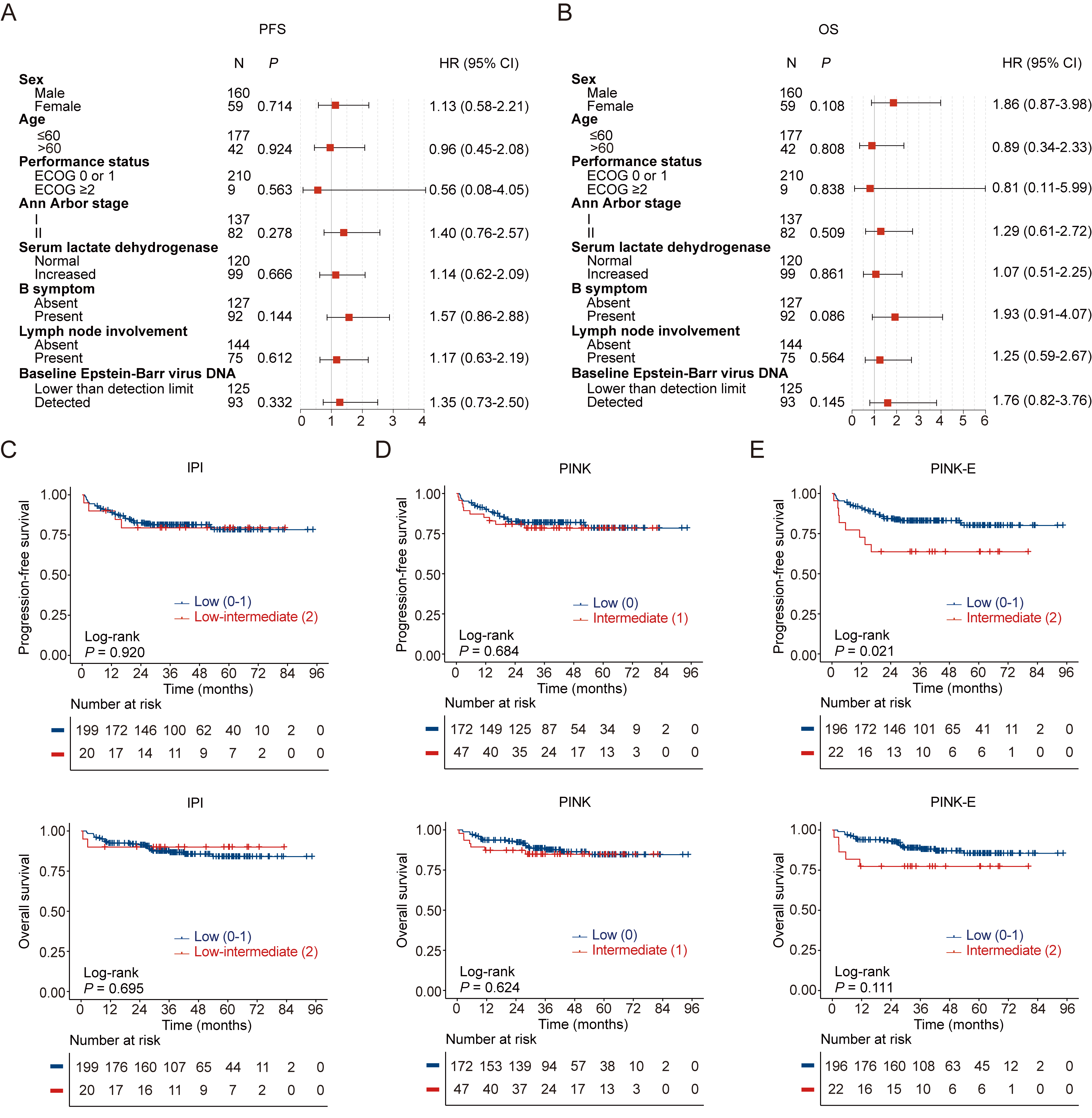
**

**Supplementary Figure S2. Clinical prognostic factors in early-stage NKTCL.**

(A and B) Forest plots showed the hazard ratio and 95% CI from univariate Cox regression models of clinical factors according to PFS (A) and OS (B), respectively.

(C-E) PFS and OS in early-stage NKTCL patients according to risk groups stratified by IPI (C), PINK (D), and PINK-E (E), respectively. *P* values were calculated by log-rank test.


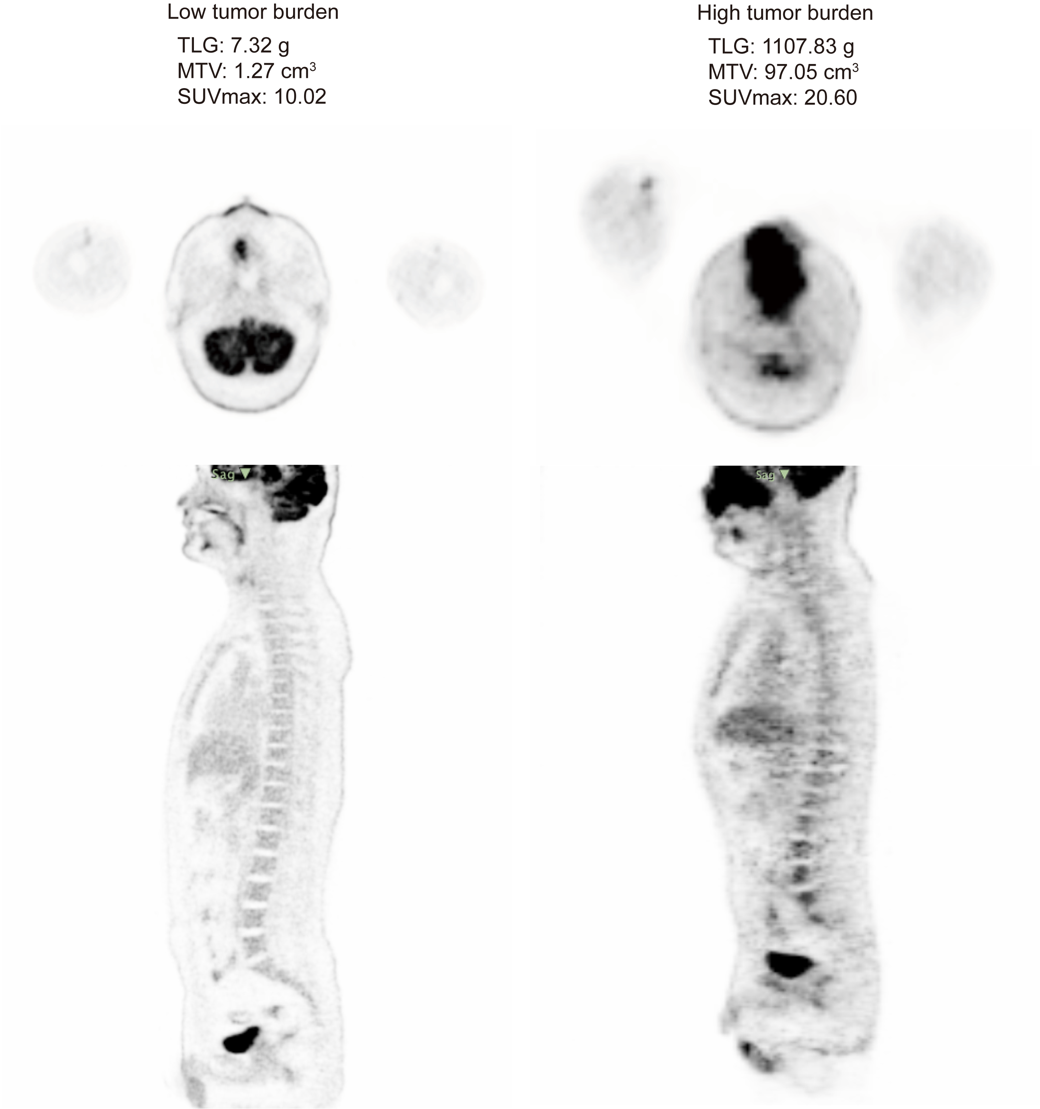


**Supplementary Figure S3. Representative PET images with low vs. high tumor burden.**

**
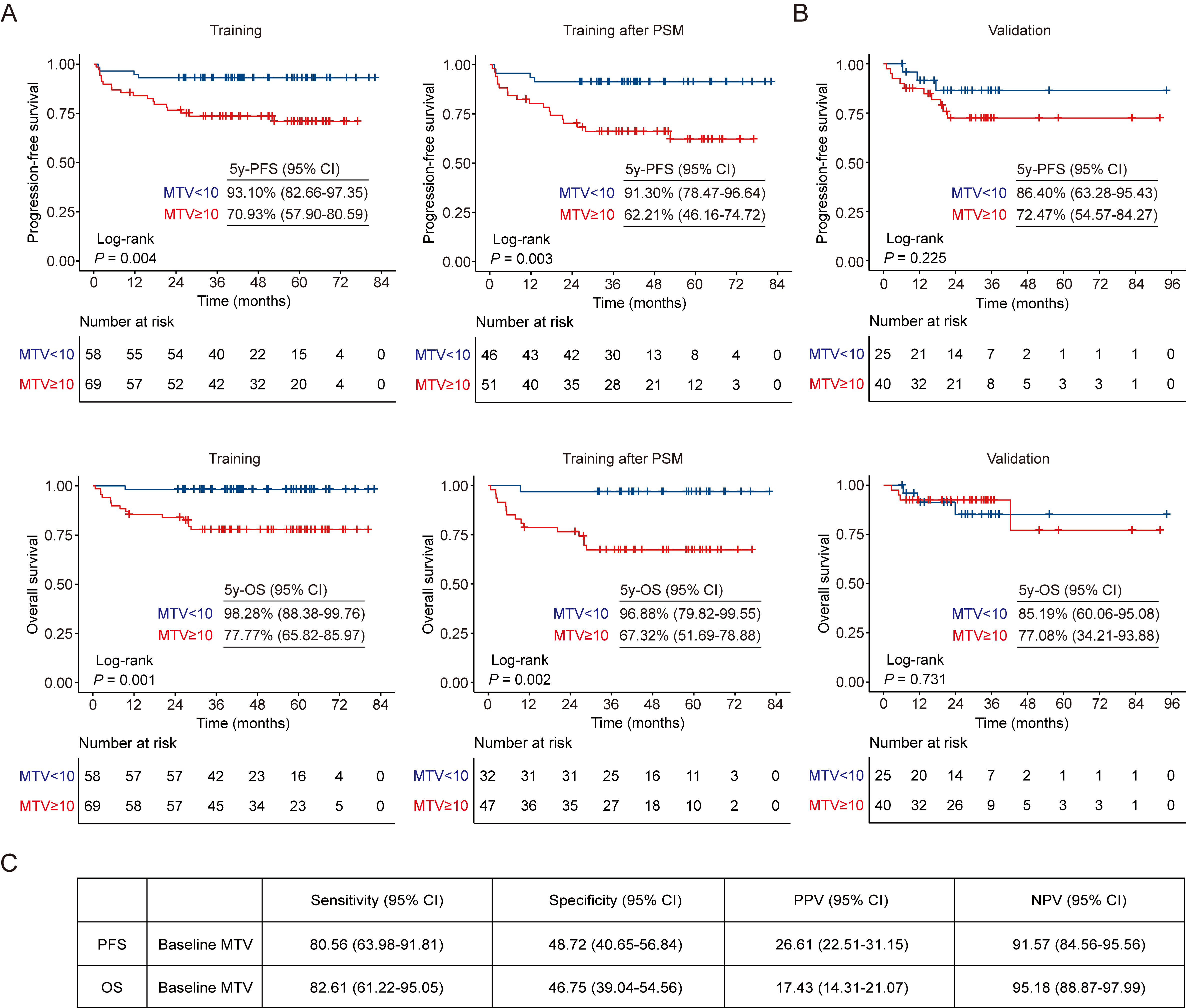
**

**Supplementary Figure S4. Predictive value of baseline MTV.**

(A and B) PFS and OS according to baseline MTV with a cutoff of 10 cm^3^ in the training cohort, training cohort after PSM (A), and validation cohort (B), respectively.

(C) Prognostic power of baseline MTV according to PFS and OS, respectively.


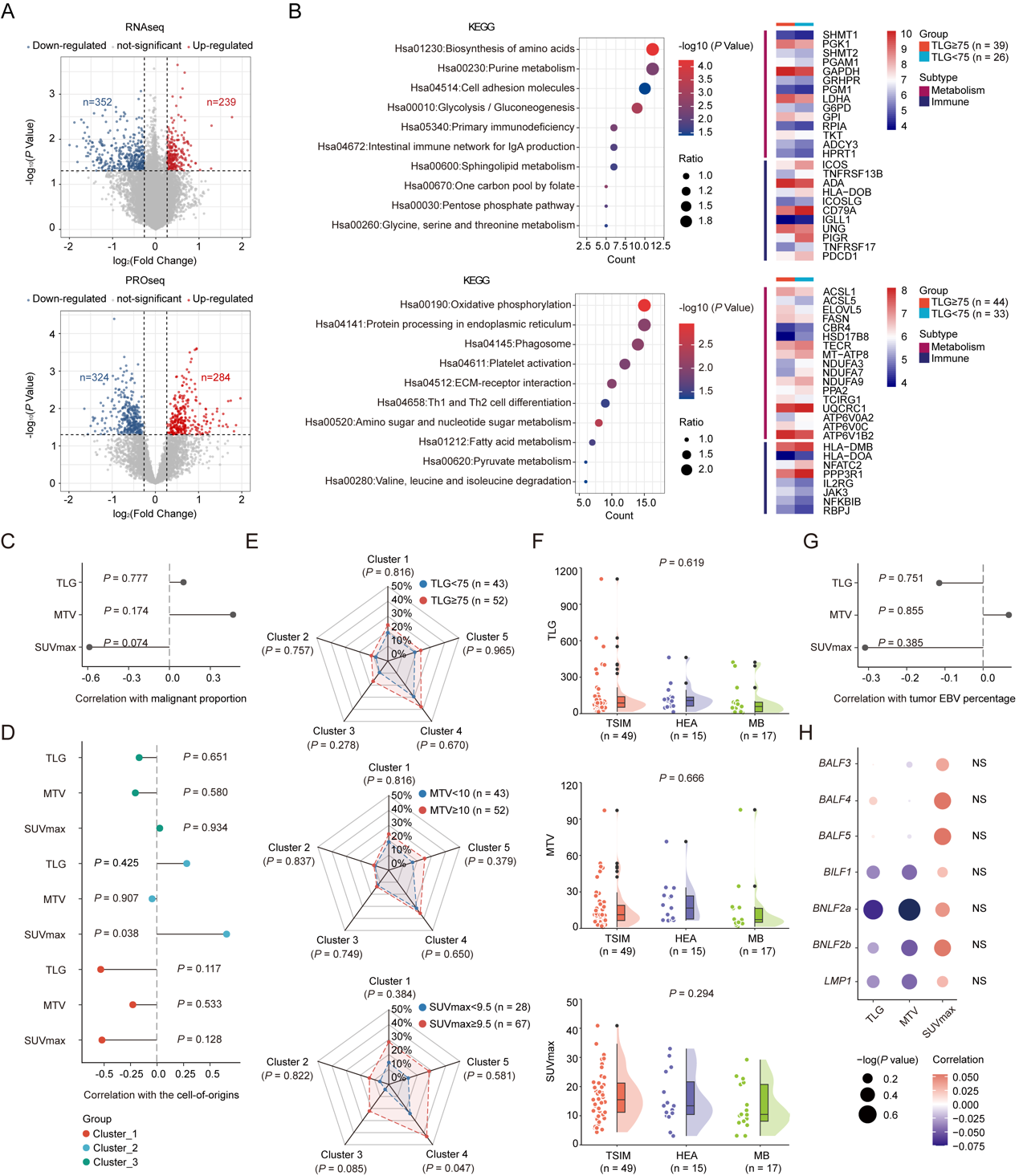


**Supplementary Figure S5. Biological features of radiomic biomarkers.**

(A) Differentially expressed genes (DEGs) in TLG high and low groups were identified based on RNA-seq (n = 65) and proteomic data (n = 77) with threshold of *P* value >0.05 and >1.2-fold change.

(B) KEGG pathway enrichment analysis and heatmap analysis of DEGs.

(C and D) Correlation of radiomic markers and proportion of malignant cells (C) and the cell-of-origins, including cluster 1-3 (D).

(E) Mutation frequency among 5 functional clusters in low TLG (< 75 g, n = 43) and high TLG (≥ 75 g, n =52) groups, in low MTV (< 10 cm^3^, n = 43) and high MTV (≥ 10 cm^3^, n =52) groups, and in low SUVmax (< 9.5, n = 28) and high SUVmax (≥ 9.5, n =67) groups, respectively. *P* values were calculated with χ2 or Fisher's exact test.

(F) Comparison of baseline TLG, MTV and SUVmax according to the molecular subtypes based on 81 NKTCL patients. *P* values were compared using ANOVA. Data were represented as mean ± SEM.

(G and H) Correlation of radiomic markers and tumor EBV percentage (G) and viral gene expression (H).


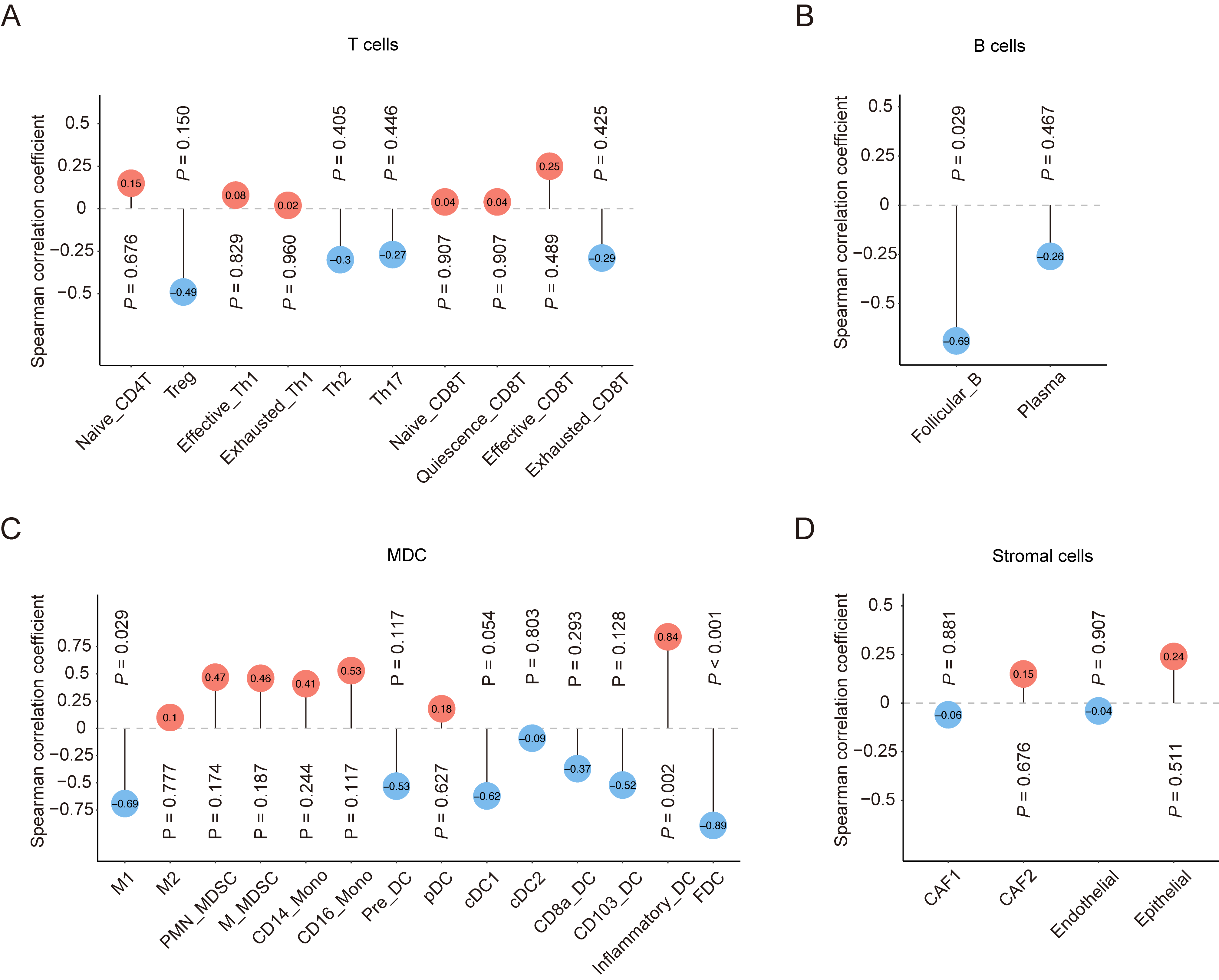


**Supplementary Figure S6. Baseline TLG and tumor microenvironment.**

(A-D) Correlations of baseline TLG and T cells (A), B cells (B), myeloid dendritic cell (MDC) (C) and stromal cells (D) based on scRNA-seq data of NKTCL patients (n = 10).


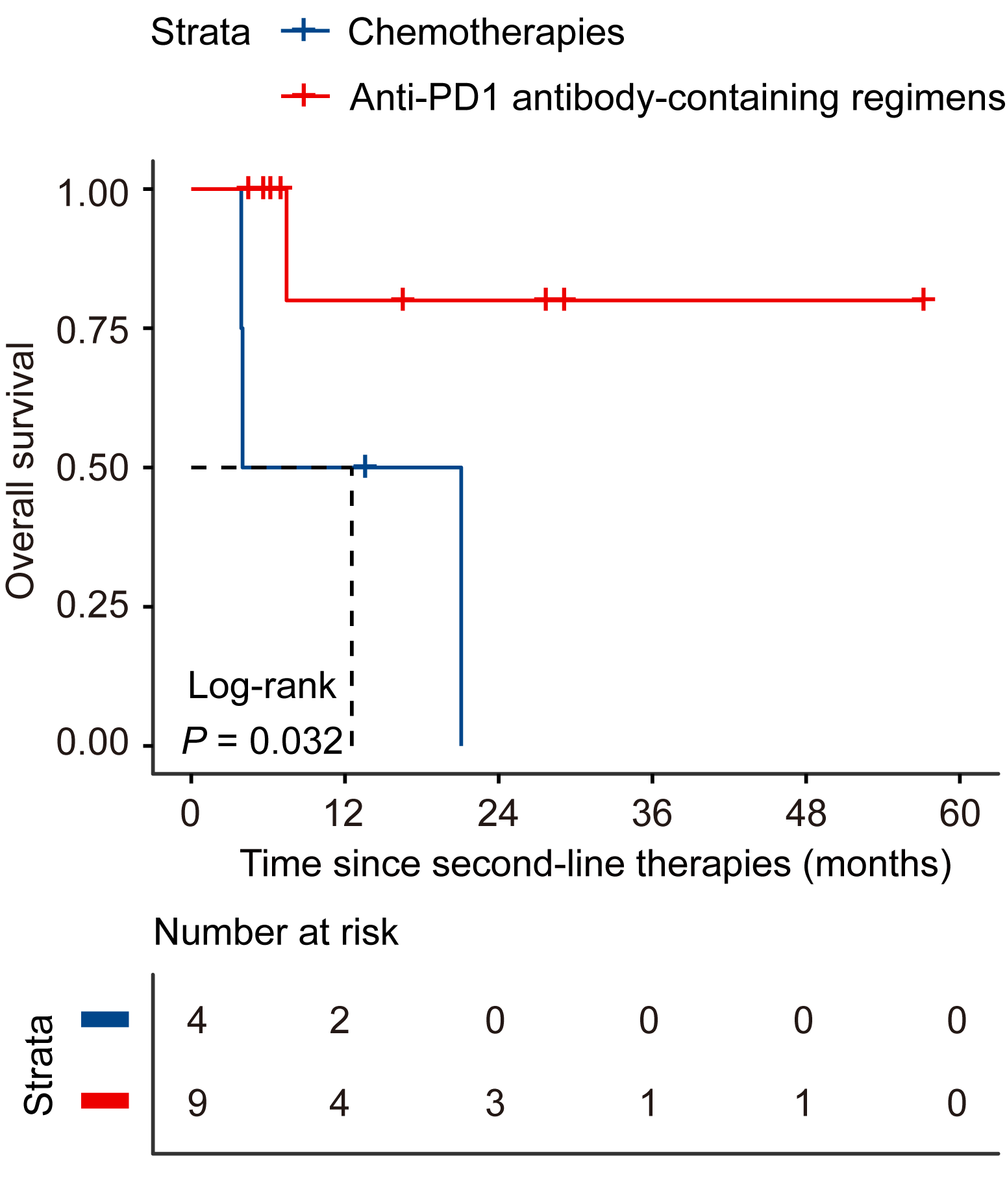


**Supplementary Figure S7. Survival outcomes in refractory/relapsed patients with high baseline TLG receiving second-line therapies.**

OS stratified by regimens (anti-PD1 antibody-containing regimens vs. chemotherapies) in refractory/relapsed patients with high baseline TLG, respectively. *P* values were calculated by log-rank test.
